# Comparison of the Intestinal Pharmacokinetics of Two Different Florfenicol Dosing Regimens and Its Impact on the Prevalence and Phenotypic Resistance of *E. coli* and *Enterococcus* over Time

**DOI:** 10.3390/microorganisms9091835

**Published:** 2021-08-30

**Authors:** Jennifer L. Halleran, Ryker Minch, Hannah J. Slyvester, Megan E. Jacob, Timo Prange, Ronald Baynes, Derek M. Foster

**Affiliations:** 1 Department of Population Health and Pathobiology, College of Veterinary Medicine, NC State University, Raleigh, NC 27607, USA; rminch@ncsu.edu (R.M.); hannahjslyvester@gmail.com (H.J.S.); megan_jacob@ncsu.edu (M.E.J.); Ronald_Baynes@ncsu.edu (R.B.); dmfoster@ncsu.edu (D.M.F.); 2 Department of Clinical Sciences, College of Veterinary Medicine, NC State University, Raleigh, NC 27607, USA; tprange@ncsu.edu

**Keywords:** antimicrobials resistance, cattle, gastrointestinal pharmacokinetics

## Abstract

In order to mitigate the food animal sector’s role in the growing threat of antimicrobial resistance (AMR), the World Health Organization (WHO) suggests the use of lower tier antimicrobials, such as florfenicol. Florfenicol has two dosing schemes used to treat primarily bovine respiratory disease. In this study, the objective was to characterize the plasma and gastrointestinal pharmacokinetics of each dosing regimen and assess the effect of these dosing regimens on the prevalence of resistant indicator bacteria over time. Twelve steers underwent abdominal surgery to facilitate the placement of ultrafiltration probes within the lumen of the ileum and colon, as well as placement of an interstitial probe. Following surgery, cattle were dosed with either 20 mg/kg IM every 48 h of florfenicol given twice (n = 6) or a single, subcutaneous dose (40 mg/kg, n = 6). Plasma, interstitial fluid, gastrointestinal ultrafiltrate, and feces were collected. Pharmacokinetic analysis demonstrated high penetration of florfenicol within the gastrointestinal tract for both the high and low dose group (300%, 97%, respectively). There was no significant difference noted between dosing groups in proportion or persistence of phenotypically resistant bacterial isolates; however, the percent of resistant isolates was high throughout the study period. The recommendation for the use of a lower tier antimicrobial, such as florfenicol, may allow for the persistence of co-resistance for antibiotics of high regulatory concern.

## 1. Introduction

In the United States, the antimicrobial resistance (AMR) crisis is ever growing; more than 2.8 million antibiotic resistance infections occur each year, with more than 35,000 human mortalities [1]. Human exposure to antimicrobial resistant bacteria is multifactorial, involving human, animal, and environmental sources. To combat the growing AMR threat, the World Health Organization (WHO) has classified the 35 classes of antimicrobials according to importance and priority use in human medicine [2]. Based upon these antimicrobial classifications, the WHO has put forth recommendations regarding antimicrobial use in food producing species to decrease the food animal sector’s contribution to the rise in AMR [2]. One such recommendation is to use antimicrobials of lower importance and priority in human medicine, a “lower tier” antibiotic. Florfenicol is an example of a “lower tier” antibiotic put forth by the WHO.

Florfenicol is a fluorinated analog of thiamphenicol and chloramphenicol [3]. Florfenicol inhibits bacterial protein synthesis by binding to the 50S ribosomal subunit [4]. In veterinary medicine, injectable florfenicol was first approved by the FDA in 1996 for the treatment of bovine respiratory pathogens [3]. It is currently approved for the treatment and control of bovine respiratory disease and foot rot. Resistance in bacteria of human origin to chloramphenicol is not a concern because of its rare use due to its association with aplastic anemia; therefore, resistance to florfenicol alone is not likely highly consequential. However, with the increasing use of florfenicol in veterinary medicine, resistance patterns against florfenicol and other antibiotic classes have been recognized, including antibiotic classes of high regulatory concern. For example, linezolid resistant human strains of *Enterococcus* spp. may carry plasmids with co-resistance genes to florfenicol [5]. The *cfr* gene (named for chloramphenicol and florfenicol resistance) also mediates resistance to both linezolid and florfenicol in methicillin-resistant staphylococci [6]. Thus, the use of florfenicol could co-select for resistance to other important antimicrobials. In *E. coli*, common resistance patterns include resistance to florfenicol, ampicillin, ceftiofur, and tetracycline [3,7].

There are two injectable dosing regimens of florfenicol in veterinary medicine, a lower intramuscular dose (20 mg/kg IM) given twice, 48 h apart, or a single, higher subcutaneous injection (40 mg/kg SC). Each dosing regimen has its own respective withdrawal interval; 28 days following the second administration of the intramuscular injection and 38 days following the subcutaneous injection. A relationship exists between sub-therapeutic drug concentrations and the amplification of a resistant sub-population of bacteria. It is believed that when dosing lower concentrations of antimicrobials for a longer period of time, maximum proliferation of a resistant sub-population of bacteria occurs; this is compared to higher concentrations of the same antimicrobial administered once early in the disease process [8]. This phenomenon is known as the inverted U plot, and it suggests the best course of therapy, in terms of mitigating a resistant population of bacteria, is to administer a single high dose of an antibiotic early in the disease process [8]. With parenteral administration of antibiotics, it is likely that enteric sub-therapeutic drug concentrations will occur; this will exert pressure on the intestinal bacteria, and allow for a resistant sub-population of bacteria to amplify, which could be transferred to humans.

In 1996, the FDA, USDA, and CDC established the National Antimicrobial Resistance Monitoring Systems (NARMS) as a surveillance program to monitor antimicrobial susceptibility of enteric pathogens in humans, animals, and retail meats. NARMS also became involved in epidemiology studies assessing risk factors and clinical case outcomes of infectious agents with specific resistance patterns. In the animal sector, *E. coli* and *Enterococcus* were utilized as marker organisms to gauge antimicrobial susceptibility and resistance patterns that could be passed along to humans.

The overall objective of this study was to (1) characterize the gastrointestinal pharmacokinetics of florfenicol and (2) compare the prevalence of resistant bacterial isolates over time between the two labeled dosing regimens for florfenicol in cattle. In order to assess the human implications associated with florfenicol administration, human breakpoint concentrations for *E. coli* and *Enterococcus* and their associated studied antibiotic were utilized. The overall hypothesis for the study was the prevalence of resistant bacterial isolates would be higher and persist longer in steers administered the repeated, lower dose of florfenicol. A secondary hypothesis was that the gastrointestinal concentrations of florfenicol would be high, leading to a subsequent rise in resistant bacterial isolates.

## 2. Materials and Methods

### 2.1. Animals, Surgical Procedure and Treatment

This study was approved by North Carolina State University’s Institutional Animal Care and Use Committee. Twelve healthy 6–7 month old steers (153.3–251.8 kg) were enrolled in the study. The study size was based on previous gastrointestinal pharmacokinetic studies in order to demonstrate differences between the two dosing regimens [9,10]. They were judged healthy by a physical exam on presentation and had no previous documentation of any antimicrobial administration. After a three-day period of acclimation, the steers underwent gastrointestinal surgery to facilitate the placement of ultrafiltration probes, as previously described [10,11]. The steers were clipped and sterilely prepped on their right flank. A distal paravertebral block with lidocaine was used to provide anesthesia to the right paralumbar fossa. A linear vertical incision was made. Upon entering the abdomen, the cecum was found. Proximal to the cecum, a segment of ileum was exteriorized through the abdominal incision. A small incision was made in the ileum and the collecting loops of the ultrafiltration probe (UF-3-12, BAS; Bioanalytical Systems, West Lafayette, IN, USA) were introduced into the lumen via an introducer needle. A purse-string suture pattern was applied to secure the ultrafiltration probe. The same procedure was performed to place an ultrafiltration probe into the lumen of the spiral colon. The free ends of the ultrafiltration probes were brought out of the abdomen near the abdominal incision. The flank incision was closed in a routine manner. At the time of surgery, steers received either intravenous flunixin meglumine (2 mg/kg, Banamine^®^, Merck Animal Health) or transdermal flunixin meglumine (3.3 mg/kg, Banamine^®^ Transdermal, Merck Animal Health).

Twenty-four to 48 h after surgery, the steers were dosed with either 20 mg/kg florfenicol (Nuflor^®^, Merck Animal Health) intramuscularly every 48 h (n = 6) twice or a single 40 mg/kg subcutaneous dose (n = 6). The steers were housed in pairs (one from each treatment group) and fed grass hay with supplemental grain and free access to water for the duration of the study. Feces and dirty shavings were removed to prevent cross-contamination by means of ingesting potential excreted drug.

### 2.2. Collection of Plasma

To facilitate blood collection, prior to surgery, an intravenous jugular catheter (Angiocath^TM^, Becton Dickinson Infusion Therapy Systems Inc., Sandy, UT, USA) was placed. Blood was obtained at 0, 15, and 30 min, and 2, 4, 8, 12, 24, 36, 48 h, 48 h 15 min, 48 h 30 min, 49, 50, 52, 56, 60, 72, 84, 96, 120, 144, and 168 h. The blood was spun down at 1000× *g* for 10 min. The plasma was transferred to cyrovials and stored at −80 °C until analysis.

### 2.3. Collection of Interstitial Fluid and Intestinal Ultrafiltrate

After the completion of surgery with properly placed ultrafiltration probes, the free ends of the ultrafiltration probes from the ileum and spiral colon were connected to a 3 mL evacuated tube with no additive (Vacutainer R, Becton-Dickinson). This was done by connecting the probe onto a needle of the vacuum vial needle holder at the end of the tubing. The external tubing was sutured in place along the flank. Intestinal ultrafiltrate was collected by changing the tubes at the following time points: 0, 2, 4, 6, 8, 12, 24, 26, 28, 30, 32, 36, 48, 72, and 96 h post high-dose drug administration, and additionally 50, 52, 54, 56, 60, 74, 76, 78, 80, and 84 h post low-dose drug administration. The fluid was collected into cyrovials and stored at −80 °C.

An ultrafiltration probe was also used to collect interstitial fluid (ISF); this probe was placed in the subcutaneous space at the withers. The free end of the probe was connected to an evacuated tube in the same fashion and sutured along the back. The evacuated tube was changed at 0, 2, 4, 8, 12, 24, 36, 48, 50, 52, 56, 60, 72, 84, 96, 120, 144, and 168 h. The fluid was transferred into cyrovials and stored at −80 °C.

### 2.4. Drug Concentration Analysis

The samples were analyzed for florfenicol using reverse phase high-pressure liquid chromatography (HPLC) with ultraviolet (UV) absorbance detection for plasma samples and fluorescence detection for tissue fluids with methods previously used and validated in our laboratory [11], which had been modified from others [12,13]. The ISF, colon, and ileum fluid samples were analyzed directly (without extraction) because the fluid already represented a protein-free ultrafiltrate. A validation of the assay was performed by fortifying blank plasma or other fluids (phosphate-buffered saline, HyClone, VWR, Radnor, PA, USA) with a florfenicol analytical reference standard (Florfenicol, Sigma-Aldrich, St. Louis, MO, USA) to produce concentrations for a calibration curve and quality control (QC) samples. Blank (control) samples were analyzed to measure background noise and verify that there were no interfering peaks in the chromatograms for the retention time of interest. The accuracy of the method was approximately 100% for all the spiked samples and they fell within our threshold cutoff of 15%. The limit of detection for plasma was 0.005 µg/mL and the limit of quantification was 0.01 µg/mL. For the ISF and intestinal ultrafiltrate, the limit of detection was 0.05 µg/mL and the limit of quantification was 0.1 µg/mL.

### 2.5. Pharmacokinetic Analysis

For the pharmacokinetic analysis, a computer program (Phoenix™ WinNonlin, V. 8.0; Certara^®^, St. Louis, MO, USA) was used to determine the values for pharmacokinetic parameters. Plasma, ISF, and intestinal drug concentrations were plotted on linear and semi-logarithmic graphs for analysis and for visual assessment of the best model for pharmacokinetic analysis. Specific models (e.g., one, two, etc. compartments) were determined for best fit based on visual analysis for goodness of fit and by visual inspection of residual plots for the plasma samples.

Secondary parameters calculated included the peak concentration (Cmax), time to peak concentration (Tmax), area under the plasma-concentration vs. time profile (AUC), and the elimination half-life (t ½). The ISF and intestinal ultrafiltrate samples were corrected for lag time. This was determined by measuring the volume of the ultrafiltration tube and dividing by the flow rate (μL/min) for each fluid type. The average lag time per fluid type was then used to correct for the actual sample collection time.

The relative drug transfer from the plasma compartment to the ISF and intestinal fluids was measured by calculation of a penetration factor. The penetration factor was determined by the ratio of AUC for the tissue fluid (ISF, ileum, or colon) to the AUC for plasma as follows:

Penetration Factor = AUC Tissue Fluid or ISF/AUC Plasma.

Individual Wilcoxon ranked sum tests were used to determine significance between dosing groups for the following pharmacokinetic parameters for all fluid types (plasma, interstitial fluid and intestinal ultrafiltrate): AUC, half-life, C_MAX_, T_MAX_, and intestinal penetration.

### 2.6. Collection of Feces

Feces was collected manually from the rectum. Time points for feces collection were 0, 12, 24, 36, 48, 60, 72, 84, 96, 120, 144, 168, and 192 h, and then weekly post first drug administration until day 38. The samples were placed into bags (Whirlpak^®^, Nasco, Fort Atkinson, WI, USA) and stored on ice until delivered to the laboratory.

### 2.7. Quantification of E. coli and Enterococcus from Feces

One gram of feces was weighed and placed into either 9 mL of EC broth (Oxoid Ltd., Basingstoke, Hampshire, UK) or 9 mL of Phosphate Buffered Saline (PBS, Fisher Bioreagents, Waltham, MA, USA) for quantification of *E. coli* and *Enterococcus*, respectively, at all time points. The samples were vortexed and subsequently serially diluted 10-fold into sterile phosphate buffer. The diluted samples were plated in triplicate (100 μL each) on selective media; *E. coli* concentrations were determined on Difco^TM^ MacConkey Agar (Becton, Dickinson and Company, Sparks, MD, USA) and *Enterococcus* concentrations were determined on Difco^TM^ m-Enterococcus Agar (Becton, Dickinson and Company, Sparks, MD, USA). The MacConkey Agar plates were incubated overnight at 37 °C, while the *Enterococcus* plates were incubated for 48 h at 37 °C. Dilutions that yielded colony counts between 30–300 were used; the three replicates were averaged to determine the quantity of both *E. coli* and *Enterococcus* at each time point. From the plates that were used to determine the quantity of *E. coli* and *Enterococcus*, eight isolates were randomly selected and were streaked for isolation onto Columbia agar with 5% sheep blood (Remel, Lenexa, KS, USA) and incubated overnight at 37 °C. After incubation, each suspected *E. coli* isolate was indole tested (Indole Reagent Kovacs, Remel, Lenexa, KS, USA). Each isolate was then stored in a cryogenic vial containing LB Broth (Sigma-Aldrich, St. Louis, MO, USA) supplemented with 25% glycerol (Fisher BioReagents^TM^, Fisher Scientific). They were vortexed and frozen at −80 °C as a pure growth. *Enterococcus* isolates were speciated using MALDI-TOF and frozen as stated above.

### 2.8. Quantification of E. coli and Enterococcus from Feces on Antibiotic Infused Media

**Figure 1:**
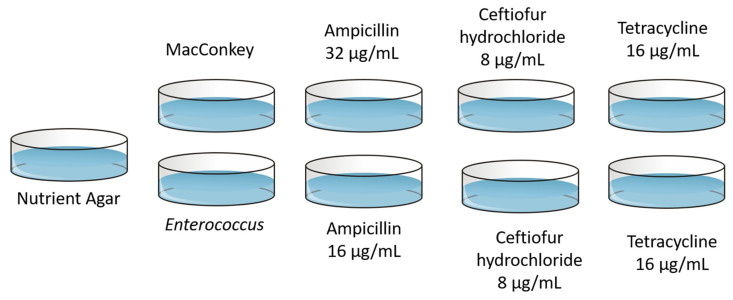
The experimental design quantifying *E. coli* or *Enterococcus* on MacConkey agar or m-Enterococcus agar, respectively, and media containing tetracycline, ceftiofur hydrochloride or ampicillin. Nutrient agar was utilized as the positive control.

In addition to being plated on selective agar, 100 μL from each dilution of EC and PBS was also plated onto nutrient agar (Difco^TM^, BD), MacConkey containing tetracycline (16 μg/mL, Sigma-Aldrich (SA), Saint Louis, MO, USA), ceftiofur hydrochloride (8 μg/mL, SA), or ampicillin (32 μg/mL, SA), or m-Enterococcus containing tetracycline (16 μg/mL), ceftiofur hydrochloride (8 μg/mL), or ampicillin (concentration of 16 μg/mL). Plates were incubated and counted; isolates were characterized and saved as described above. Isolates were considered resistant if they grew on their respective media containing antibiotics. This design is shown in Figure 1. The antibiotic concentrations utilized in the plates are the CLSI breakpoints for the bacteria–antibiotic combination being tested. This was done to assess bacterial growth and resistance of human implication following veterinary administration of florfenicol. The MIC interpretive CLSI breakpoints can be found in Table 1.

### 2.9. Calculation of Prevalence and Statistical Analysis

Resistant bacterial isolates were defined as bacterial organisms found to grow on the antibiotic infused plates; the infused antibiotic concentrations are the CLSI breakpoint for the antibiotic-bacteria pair associated with resistance. The concentration of *E. coli* and *Enterococcus* was determined for each fecal sample over time. In addition, to determine the proportion of bacterial isolates phenotypically demonstrating resistance to antibiotics, we divided the concentration of either *E. coli* or *Enterococcus* obtained on the selective agar plus antibiotics at each time point by the concentration recovered on the selective media alone.

Predetermined individual student T-tests were performed with Bonferonni correction. This was conducted to prevent inflation of type 1 error while being able to use a model that may lead to predictability. The individual t tests conducted for the concentration of *E. coli* and *Enterococcus* obtained for the subcutaneous dose at Day 0 to Day 38 and for the intramuscular dose at Day 0 to Day 28 was performed; for the proportion of resistant isolates, the subcutaneous dosing group was compared at Day 0 to Day 38, the intramuscular group was compared at Day 0 to Day 28. With Bonferonni correction, significance was indicated as a *p*-value of less than 0.0125.

## 3. Results

### 3.1. Pharmacokinetic Analysis

**Figure 2:**
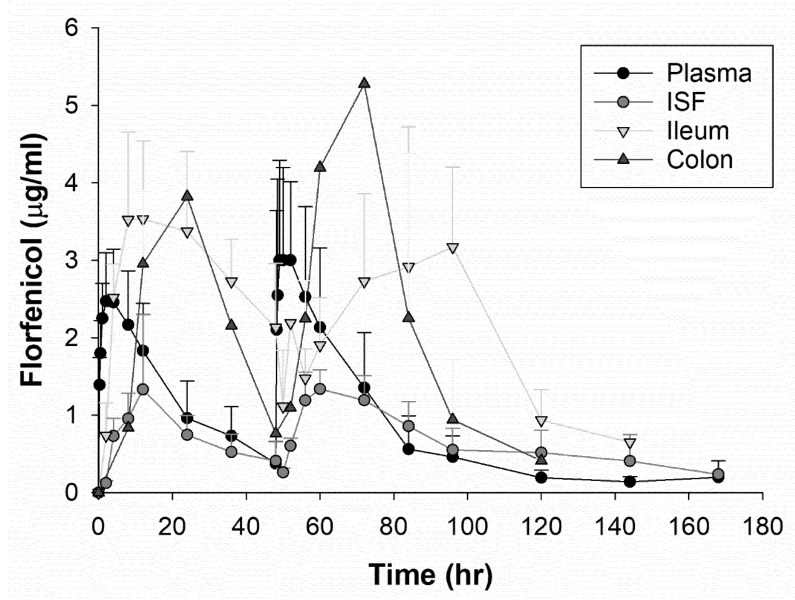
The drug concentration of florfenicol over time for plasma, interstitial fluid, ileum, and colon ultrafiltrate for the steers administered 20 mg/kg of florfenicol intramusculary every 48 h twice. The points represent the means with standard error bars.

**Figure 3:**
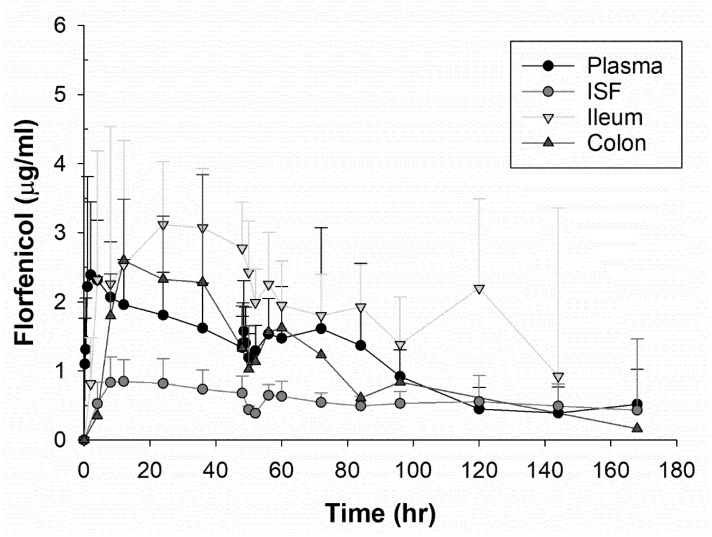
The drug concentration of florfenicol over time for plasma, interstitial fluid, ileum, and colon ultrafiltrate for the steers administered 40 mg/kg of florfenicol subcutaneously once. The points represent the means with standard error bars.

After comparing multiple one and two compartment models and a non-compartmental analysis, an extravascular, one compartment model with first order input and elimination with no lag time was the best fit for the plasma data based upon residual error plots, Akaike information criteria (AIC), and observation of observed vs. predictive plots. A comparison of the drug concentration of each fluid type per dosing group can be observed in Figure 2 and Figure 3. For the plasma compartmental analysis, there was a statistically significant difference in elimination half-life (*p* = 0.005) and AUC (*p* = 0.005) between the dosing groups, both being increased in the higher, single dose group. A non-compartmental analysis was used for the ISF and intestinal ultrafiltrate because there were insufficient samples to fit a model to these data. The geometric means and geometric CV% for selected pharmacokinetic parameters for each dosing group are shown in Table 2 and Table 3. For the ileum ultrafiltrate, a statistically significant difference was observed for T_MAX_ (*p* = 0.01) that was higher in the low dose group.

### 3.2. Bacterial Quantification and Proportion of Resistant Isolates

**Figure 4:**
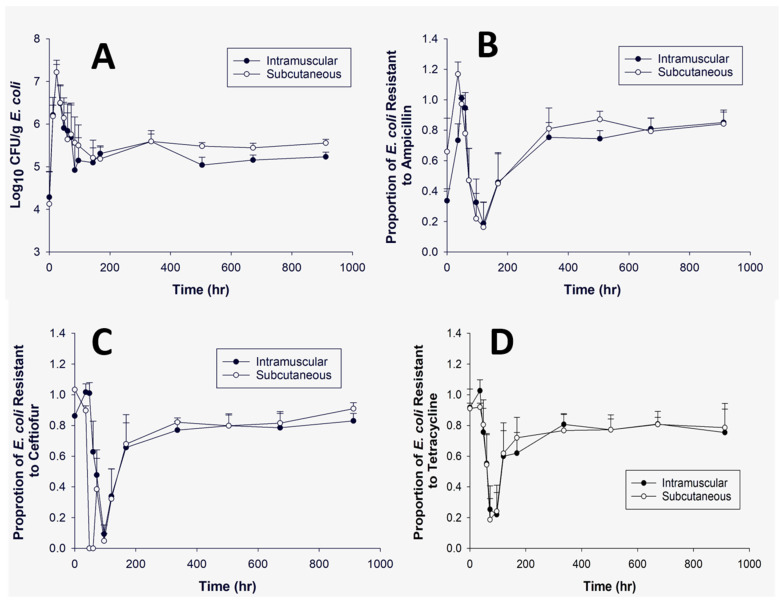
This panel represents *E. coli* isolates grown over time with no antibiotic (**A**), ampicillin (32 μg/mL) (**B**), ceftiofur (8 μg/mL) (**C**), or tetracycline (16 μg/mL) (**D**). The dark circle presents the intramuscular dosing group and the open circle represents the subcutaneous dosing group over a 912 h time period. Panel (**A**) demonstrates the log_10_ CFU/g of *E. coli*. Panels (**B**–**D**) show the proportion of *E. coli* demonstrating phenotypic resistance based on human breakpoints. Bars represent the standard error of the mean. There were no significant differences noted for each dosing group per plain and antibiotic infused plates at the predetermined time points.

**Figure 5:**
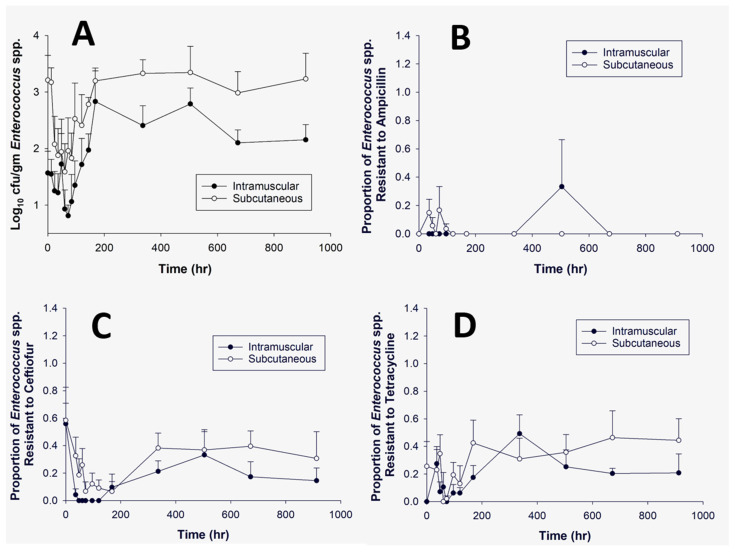
This panel represents *Enterococcus* isolates grown over time on no antibiotic (**A**), ampicillin (16 μg/mL) (**B**), ceftiofur (8 μg/mL) (**C**), or tetracycline (16 μg/mL) (**D**). The dark circle presents the intramuscular dosing group and the open circle represents the subcutaneous dosing group over a 912-h time period. Panel (**A**) demonstrates the log_10_ CFU/g of *Enterococcus*. Panels (**B**–**D**) show the proportion of *Enterococcus* demonstrating phenotypic resistance based on human breakpoints. Bars represent the standard error of the mean. There were no significant differences noted for each dosing group per plain and antibiotic infused plate at the predetermined time points.

Colonies of either *E. coli* or *Enterococcus* were obtained on selective media without antibiotics and nutrient agar at all time points. The average concentration (log_10_ CFU/g) of *E. coli* and *Enterococcus* can be observed in Figure 4A and Figure 5A, respectively. There appeared to be an initial increase in *E. coli* (0–24 h) and a decrease (0–24 h) in *Enterococcus* immediately after administration of florfenicol. Figure 4B–D demonstrates the average proportion of *E. coli* isolates phenotypically resistant to ampicillin, ceftiofur or tetracycline. At time point zero, there was a high proportion of resistant isolates observed for both ceftiofur and tetracycline; these both then decrease. Figure 5B–D demonstrates the proportion of resistant *Enterococcus* for ampicillin, ceftiofur or tetracycline. There was little *Enterococcus* growth on any of the ampicillin infused plates. There were no significant differences noted at the predetermined individual time points when comparing within or across treatment groups for either *E. coli* or *Enterococcus* spp. (Table 4 and Table 5).

## 4. Discussion

In this study, the plasma, ISF and gastrointestinal drug concentrations of florfenicol were characterized between two different dosing regimens, a lower intramuscular dose administered twice (48 h apart) and a higher, single subcutaneous dose. In addition, indicator enteric organisms, *E. coli* and *Enterococcus*, were grown on selective media, as well as media containing antibiotics to assess the proportion of isolates demonstrating phenotypic resistance to common drugs with co-resistance to florfenicol.

For the plasma pharmacokinetic drug analysis, there was a significant difference between dosing groups in the AUC and T ½, with both being increased in the higher, single dose group. The difference observed between each dose is likely related to the 2× higher dose. This is expected to increase the total drug absorbed, which is reflected in the AUC. The longer T ½ from a higher dose is likely caused by slow absorption from a higher volume of injection. Florfenicol can be irritating to tissues and a higher subcutaneous administration volume could lead to more erratic and prolonged absorption, which then could lead to a longer T ½. In pharmacokinetic terms, this is known as the “flip-flop” effect, where the rate of absorption influences the terminal slope of the plasma vs. time profile [16]. Previous studies have assessed florfenicol plasma drug concentrations in cattle after parenteral administration [11,12,17,18]. In the study conducted by Gilliam et al., florfenicol was administered in a digital vein for means of regional limb perfusion at a significantly lower dose (2.2 mg/kg). Due to the route and dosage administered, it is difficult to draw comparisons with our study. In the studies conducted by Foster et al. and Kawalek et al., a single subcutaneous dose (40 mg/kg) was administered; our study yielded similar C_MAX_ and T_MAX_ values when compared to the study conducted by Foster et al. However, in this study we observed a longer half-life (56.95 h), which was more similar to the study conducted by Kawalek et al. (51.9 h). The AUC measured in our study is higher than the values of either prior study. The reason for this difference is undetermined. The study conducted by Lobell et al. assessed florfenicol drug concentrations following a 20 mg/kg IM dose administered only once. Although the number of doses administered was different, the mean pharmacokinetic parameters were similar to our study.

Measurement of drug concentrations in plasma includes both protein bound and unbound drug. Only the free concentration is microbiologically active [19]. Free, non-protein bound drug is the best measurement of biologically available drug that would exert an antimicrobial effect on the gastrointestinal microflora. The semi-permeable ultrafiltration probe facilitates the collection of fluid that contains molecules weighing less than 30,000 Daltons. Therefore, only the free fraction of the drug is measured and ISF and gastrointestinal ultrafiltrate are appropriate fluid types to assess microbiologically active drug concentrations. In the study conducted by Foster et al. (2016), ISF florfenicol concentrations were assessed in steers receiving a single subcutaneous dose (40 mg/kg). Between the two studies, the AUC and penetration factor were similar. In this study, the penetration factor in the ISF for the high and low dose group is 97% and 70%, respectively. Florfenicol has low plasma protein binding; therefore, such a high level of diffusion across capillaries to the interstitial fluid is anticipated [9]. The ileum ultrafiltrate in the high dose group had the greatest penetration factor (302%) but was also high for the concentrations within the colon (91%). For the low dose group, the penetration factor was greatest in the colon (115%). The cause of these differences is undetermined, but degradation of the agent in the colon is a possibility. These values demonstrate that, regardless of dosing regimen, there is a substantial concentration of florfenicol present within the intestinal tract, exerting antimicrobial pressure on enteric bacteria.

Bacterial resistance to florfenicol falls into three main categories: minimizing intracellular concentrations, modification/protection of the antibiotic target, and modification of the antibiotic. Many of the genes responsible for florfenicol are found on mobile genetic elements, allowing for an increased probability of horizontal gene transfer and transfer of florfenicol resistance genes through many genera of bacteria. The presence of florfenicol resistance genes has been documented on plasmids that convey resistance to other antimicrobial classes, allowing for the potential of co-resistance to develop. In one study, florfenicol resistance was assessed in *E. coli* isolates collected from calves with diarrhea; all isolates studied were found to be resistant to at least four antimicrobials, while 77% were resistant to at least nine antimicrobials [3]. The most common resistance pattern observed in this study included resistance to florfenicol, ampicillin, ceftiofur, and tetracycline. In a second study, resistance patterns of *E. coli* were studied in the feces of healthy lactating dairy cattle [7]. Here, multidrug resistant (resistant to 3–6 antibiotics) *E. coli* was observed in 40% of the isolates. In this study, the most common resistance patterns identified were as follows: florfenicol and tetracycline (35.8%); ampicillin, florfenicol and tetracycline (14%); and florfenicol, ampicillin, tetracycline, and ceftiofur (0.45%). The prevalence of these specific resistance profiles influenced the decision for this study as to which antibiotics to assess. To understand the underlying genetic association with the phenotypic resistance detected, further genetic analysis would need to be conducted.

For this study, the drug concentrations that were included in the selective media were the breakpoint concentrations determined for either *E. coli* or *Enterococcus* using CLSI interpretations (Table 1). Although specific antimicrobials included may not be used in human medicine, they were representing drug classes and generations of drugs that are used regularly. We specifically used *E. coli* and *Enterococcus* as indicator organisms because of their strong presence in the gastrointestinal tract, their importance in the acquisition and transmission of antimicrobial resistance genes, their human clinical importance, and their repeated use in surveillance systems of public health. For either indicator organism, there was no significant difference observed in the concentration obtained per dosing group; however, there were elevated concentrations observed at the respective drug residue withdrawal interval. This indicates there is a potential for resistant bacterial isolates to enter the food chain. An incidental finding in this study was the unexpected level of ceftiofur and tetracycline resistant *E. coli* isolates prior to the start of this study. Tetracycline resistance is prevalent among bacterial isolates from cattle [20,21]. These steers had no documented administration of any previous antibiotics, indicating resistance to both tetracycline and ceftiofur may have been acquired through the environment. The increased ceftiofur resistance is alarming, as ceftiofur is an antimicrobial of high regulatory concern [2]. The increased levels of resistance seen at the onset of the study to ceftiofur and tetracycline may have prevented detection of increased resistance in response to florfenicol treatment.

In an effort to correlate the intestinal drug concentrations with the prevalence of resistance, we calculated the proportion of isolates demonstrating phenotypic resistance compared to the concentration of all *E. coli* and *Enterococcus* isolates at each time point. This was compared to the pharmacokinetic parameters. Figure 4B shows that the average proportion of presumptive resistant isolates appear to be increased in the high dose group when compared to the low dose group at the beginning of the study period. This increase was associated with a faster T_max_ in the ileum and colon in the high dose group compared to the low dose group. The C_max_ is also higher in the ileum for the high dose group. The higher C_max_ may increase selection for more resistant bacteria in the gut microflora and cross select for resistance to other antimicrobial classes, such as beta-lactams and tetracyclines.

There were several limitations to this study. First, the only bacterial species studied were either *E. coli* or *Enterococcus*. These two species were chosen as representatives of Gram-negative and Gram-positive bacterial species, respectively. They are also marker species utilized by NARMS to conduct surveillance for resistance. It has also been noted that these two species harbor and share antimicrobial resistance genes with other bacterial species [22]. In addition, the antibiotic concentrations used in the selective media represented the human break points for each of the tested antibiotics for either *E. coli* or *Enterococcus*. Observing the proportion of resistant isolates according to CLSI breakpoints was our primary objective. Finally, these was a relatively small sample size. It is undetermined if a larger sample size would have produced different results.

In conclusion, we reported both the plasma and gastrointestinal pharmacokinetics of two FDA-approved dosing regimens for florfenicol. Although some pharmacokinetic parameters were different between doses, the dosing regimen did not affect the proportion of resistant *E. coli* or *Enterococcus* isolates to ampicillin, ceftiofur, or tetracycline. We observed high concentrations of active florfenicol in the gastrointestinal tract lumen, which may select for resistant bacteria that can be shed in the feces and environment. Thus, use of this lower tier antimicrobial can select for antimicrobial resistance to other antimicrobial classes of high regulatory concern and those classified by the WHO as critically important. This suggests that there needs to be additional evaluation of the antimicrobial tiers and subsequent recommendations in order to best balance animal and public health.

## Figures and Tables

**Table 1:** The interpretive categories and MIC breakpoints (μg/mL) for both *E. coli* and *Enterococcus* and selected antimicrobials. The (*) indicates breakpoints were obtained from CLSI guidelines 2012–2013. The other breakpoints were obtained from CLSI guidelines 2021 [14,15].

	Interpretive Categories and MIC Breakpoints,	
	µg/mL	
Antimicrobial Agent	*E. coli*, R	*Enterococcus*, R
Ampicillin	≥32	≥16
Ceftiofur Hydrochloride *	≥8	≥8
Tetracycline	≥16	≥16

**Table 2:** Selected PK parameters following analysis for the steers administered 20 mg/kg florfenicol IM q48 h twice. The geometric mean and geometric CV% are provided. T_max_: time to maximum concentration; C_max_: the maximum drug concentration; AUC: area under the time–concentration curve.

		Plasma		ISF		Ileum Ultrafiltrate		Colon Ultrafiltrate	
Parameter	Unit	Geometric Mean	Geometric CV%	Geometric Mean	Geometric CV%	Geometric Mean	Geometric CV%	Geometric Mean	Geometric CV%
Tmax	h	1.82	26.53	59.91	10.42	76.35	13.42	61.27	18.57
Cmax	µg/mL	2.33	48.66	1.37	25.01	2.88	39.34	3.16	82.81
AUC	h·µg/mL	56.46	42.03	98.58	60.31	140.75	24.73	168.45	76.96
Half-Life	h	15.06	46.21	41.58	75.33	29.28	5.5	17.69	63.68
Penetration	%			69.95	63.44	97.93	21.88	115.60	78.10

**Table 3:** Selected PK parameters following analysis for the steers administered 40 mg/kg florfenicol SC once. The geometric mean and geometric CV% are provided. T_max_: time to maximum concentration; C_max_: the maximum drug concentration; AUC: area under the time–concentration curve.

		Plasma		ISF		Ileum Ultrafiltrate		Colon Ultrafiltrate	
Parameter	Unit	Geometric Mean	Geometric CV%	Geometric Mean	Geometric CV%	Geometric Mean	Geometric CV%	Geometric Mean	Geometric CV%
Tmax	h	2.76	52.33	48.32	180.71	14.63	63.52	15.48	85.09
Cmax	µg/mL	2.4	66.15	0.92	86.69	3.51	22.14	2.77	33.55
AUC	h·µg/mL	206.95	58.19	146.08	39.19	426.40	84.15	140.03	45.81
Half-Life	h	56.95	62.28	100.11	25.42	57.24	98.38	23.09	58.56
Penetration	%			97.62	68.04	302.28	92.28	91.61	25.33

**Table 4:** Predetermined Student’s T tests to observe significant differences at particular time points for *E. coli* isolates grown with or without antibiotics. There were no significant differences noted for any of the above comparisons.

		SC Day 0	SC Day 38	IM Day 0	IM Day 28	IM Day 28	IM Day 38	T Value	Probability
Treatment		N = 6	N = 6	N = 6	N = 6	N = 6	N = 6	N = 6	N = 6
No Antibiotic	Mean/Standard Error	4.13/(0.76)	5.55/(0.09)					−4.9	0.13
No Antibiotic	Mean/Standard Error			4.28/(0.59)	5.15/(0.12)			−4.88	0.13
Ampicillin	Mean/Standard Error	0.66/(0.22)	0.84/(0.08)					−0.39	0.71
Ampicillin	Mean/Standard Error			0.33/(0.07)	0.81/(0.07)			−0.16	0.88
Ampicillin	Mean/Standard Error		0.84/(0.08)				0.85/(0.08)	0.72	0.47
Ceftiofur	Mean/Standard Error	1.03/(0)	0.91/(0.03)					−0.39	0.71
Ceftiofur	Mean/Standard Error			0.86/(0)	0.78/(0.09)			−0.16	0.88
Ceftiofur	Mean/Standard Error		0.91/(0.03)				0.83/(0.05)	0.72	0.47
Tetracycline	Mean/Standard Error	0.91/(0.03)	0.79/(0.16)					−0.39	0.71
Tetracycline	Mean/Standard Error			0.92/(0.12)	0.8/(0.04)			−0.16	0.88
Tetracycline	Mean/Standard Error		0.79/(0.16)				0.75/(0.15)	0.72	0.47

**Table 5:** Predetermined Student’s T tests to observe significant differences at particular time points for *Enterococcus* isolates grown with or without antibiotics. There were no significant differences noted for any of the above comparisons.

		SC Day 0	SC Day 38	IM Day 0	IM Day 28	IM Day 28	IM Day 38	T value	Probability
Treatment		N = 6	N = 6	N = 6	N = 6	N = 6	N = 6	N = 6	N = 6
No Antibiotic	Mean/Standard Error	3.2/(0.44)	2.98/(0.38					−0.44	0.73
No Antibiotic	Mean/Standard Error			1.56/(0.36)	2.12/(0.23)			−1.67	0.35
Ampicillin	Mean/Standard Error	0	0					−0.39	0.71
Ampicillin	Mean/Standard Error			0	0			−0.16	0.88
Ampicillin	Mean/Standard Error		0				0	0.72	0.47
Ceftiofur	Mean/Standard Error	0.58/(0.12)	0.31/(0.19)					−0.39	0.71
Ceftiofur	Mean/Standard Error			0.55/(0.27)	0.15/(0.09)			−0.16	0.88
Ceftiofur	Mean/Standard Error		0.31/(0.19)				0.15/(0.09)	0.72	0.47
Tetracycline	Mean/Standard Error	0.26/(0.18)	0.44/(0.16)					−0.39	0.71
Tetracycline	Mean/Standard Error			0	0.2/(0.04)			−0.16	0.88
Tetracycline	Mean/Standard Error		0.44/(0.16)				0.21/(0.14)	0.72	0.47

## Data Availability

The authors can be contacted for any raw data.
